# Supplementary material for: Integrated treatment-decision algorithms for childhood TB: modelling diagnostic performance and costs
Source: IJTLD Open. 2026 Jan 9;3(1):31–7. doi: 10.5588/ijtldopen.25.0415 (PMC12826592; doi:10.5588/ijtldopen.25.0415)
Supplement: Supplementary file 1 [file ijtldopen25-0415_supplementarydata1.pdf]

SUPPLEMENTARY DATA

Integrated treatment-decision algorithms for childhood TB: modelling diagnostic performance and costs

Table S1: Model parameter estimates for children under 10 years of age with presumptive TB in Uganda

Table S2: Consolidated Health Economic Evaluation Reporting Standards checklist

Table S3: Diagnostic accuracy results for a cohort of 10,000 children under 10 years of age with presumptive TB

Figure S1: Probabilistic Sensitivity Analyses fixing single parameter values

Table S1. Model parameter estimates for children under 10 years of age with presumptive TB in Uganda

Clinical Parameters	Base	Range	Distribution	Reference
Prevalence of TB, PHC	0.03	0.02–0.04	Beta	(1)
Prevalence of TB, DH	0.10	0.08–0.12	Beta	(1)
Prevalence of HIV, PHC	0.05	0.04–0.06	Beta	(2)
Prevalence of HIV, DH	0.10	0.08–0.12	Beta	(2)
Proportion high-risk, PHC	0.50	0.40–0.60	Beta	(1)
Proportion high-risk, DH	0.60	0.48–0.72	Beta	(1)
Referral to DH, high risk	0.35	0.28-0.42	Beta	(1)
Referral to DH, low risk	0.20	0.16–0.24	Beta	(1)
Persistent symptoms, TB	0.80	0.64–0.96	Beta	(3, 4)
Persistent symptoms, TB negative	0.20	0.16–0.24	Beta	(3, 4)
Sensitivity of respiratory Xpert Ultra, children without HIV	0.73	0.59-0.85	Beta	(5)
Specificity of respiratory Xpert Ultra, children without HIV	0.95	0.91-0.98	Beta	(5)
Sensitivity of respiratory Xpert Ultra, children with HIV	0.66	0.47-0.82	Beta	(5)
Specificity of respiratory Xpert Ultra, children with HIV	0.97	0.95-0.99	Beta	(5)
Sensitivity of stool Xpert Ultra, children without HIV	0.56	0.43-0.70	Beta	(5)
Specificity of stool Xpert Ultra, children without HIV	0.98	0.95–0.99	Beta	(5)
Sensitivity of stool Xpert Ultra, children with HIV	0.61	0.42-0.77	Beta	(5)
Specificity of stool Xpert Ultra, children with HIV	0.97	0.94-0.98	Beta	(5)
Sensitivity of urine LAM, children with HIV	0.47	0.33–0.60	Beta	(6)
Specificity of urine LAM, children with HIV	0.76	0.57–0.96	Beta	(6)
Sensitivity of TDA-A with CXR	0.88	0.71–0.95	Beta	(7)
Specificity of TDA-A with CXR	0.37	0.15–0.67	Beta	(7)
Sensitivity of TDA-B	0.86	0.68–0.94	Beta	(7)
Specificity of TDA-B	0.30	0.13–0.56	Beta	(7)
Stool sample available	0.65	0.52–0.78	Beta	(1)
Urine sample available	0.65	0.52–0.78	Beta	(8)
Cost Parameters, I$	Base Case	Range	Distribution	Reference
Outpatient visit, PHC	$3.25	2.92–3.57	Gamma	(9)
Outpatient visit, DH	$4.56	4.11–5.02	Gamma	(9)

HIV testing	$9.09	8.18–10.00	Gamma	(10)
Urine LAM testing	$4.90	4.41–5.39	Gamma	(11)
Chest X-ray, DH	$11.37	10.23–12.50	Gamma	(12)
Mobile Chest X-ray, PHC	$16.30	14.67–17.93	Gamma	(13)
Stool collection	$1.99	1.79–2.19	Gamma	(14)
Expectorated sputum	$1.88	1.69–2.07	Gamma	(14)
Induced sputum	$27.57	24.81–30.32	Gamma	(14)
Gastric aspirate	$5.21	4.69–5.73	Gamma	(14)
Sample transport	$1.63	1.47–1.79	Gamma	(15)
Stool processing	$4.54	4.08–4.99	Gamma	(16)
Xpert Ultra testing	$21.86	19.67–24.04	Gamma	(15)
Cost of TB treatment, 6 months	$362.41	243.61–527.82	Gamma	(17)

Legend for abbreviations: chest X-ray (CXR), district hospital (DH), Human immunodeficiency virus (HIV), International dollars (I$), urine lateral flow lipoarabinomannan (LF-LAM), primary health center (PHC), Tuberculosis (TB), treatment-decision algorithm

Table S2: Consolidated Health Economic Evaluation Reporting Standards checklist

Topic	No.	Item	Location where item is reported
**Title**			
	1	Identify the study as an economic evaluation and specify the interventions being compared.	Title page
**Abstract**			
	2	Provide a structured summary that highlights context, key methods, results, and alternative analyses.	Abstract
**Introduction**			
**Background and objectives**	3	Give the context for the study, the study question, and its practical relevance for decision making in policy or practice.	Paragraph 2-3
**Methods**			
**Health economic analysis plan**	4	Indicate whether a health economic analysis plan was developed and where available.	Not done
**Study population**	5	Describe characteristics of the study population (such as age range, demographics, socioeconomic, or clinical characteristics).	Methods, Paragraph 1
**Setting and location**	6	Provide relevant contextual information that may influence findings.	Methods, Paragraph 1
**Comparators**	7	Describe the interventions or strategies being compared and why chosen.	Methods, Paragraphs 2-3
**Perspective**	8	State the perspective(s) adopted by the study and why chosen.	Methods, Paragraph 5
**Time horizon**	9	State the time horizon for the study and why appropriate.	Methods, Paragraph 5
**Discount rate**	10	Report the discount rate(s) and reason chosen.	Methods, Paragraph 5
**Selection of outcomes**	11	Describe what outcomes were used as the measure(s) of benefit(s) and harm(s).	Methods, Paragraph 5
**Measurement of outcomes**	12	Describe how outcomes used to capture benefit(s) and harm(s) were measured.	Methods, Paragraph 5
**Valuation of outcomes**	13	Describe the population and methods used to measure and value outcomes.	Methods, Paragraph 5
**Measurement and valuation of resources and costs**	14	Describe how costs were valued.	Methods, Paragraph 5

Topic	No.	Item	Location where item is reported
**Currency, price date, and conversion**	15	Report the dates of the estimated resource quantities and unit costs, plus the currency and year of conversion.	Methods, Paragraph 5
**Rationale and description of model**	16	If modelling is used, describe in detail and why used. Report if the model is publicly available and where it can be accessed.	Methods, Paragraph 3, Figure 1
**Analytics and assumptions**	17	Describe any methods for analysing or statistically transforming data, any extrapolation methods, and approaches for validating any model used.	Methods, Paragraph 4-6
**Characterising heterogeneity**	18	Describe any methods used for estimating how the results of the study vary for subgroups.	Methods, Paragraph 5
**Characterising distributional effects**	19	Describe how impacts are distributed across different individuals or adjustments made to reflect priority populations.	Not done
**Characterising uncertainty**	20	Describe methods to characterise any sources of uncertainty in the analysis.	Methods, Paragraph 6
**Approach to engagement with patients and others affected by the study**	21	Describe any approaches to engage patients or service recipients, the general public, communities, or stakeholders (such as clinicians or payers) in the design of the study.	Not done
**Results**			
**Study parameters**	22	Report all analytic inputs (such as values, ranges, references) including uncertainty or distributional assumptions.	Supplemental Table 1
**Summary of main results**	23	Report the mean values for the main categories of costs and outcomes of interest and summarise them in the most appropriate overall measure.	Results, Tables 2-3
**Effect of uncertainty**	24	Describe how uncertainty about analytic judgments, inputs, or projections affect findings. Report the effect of choice of discount rate and time horizon, if applicable.	Results, Figure 2, Figure 3, Supplemental Figure 1
**Effect of engagement with patients and others affected by the study**	25	Report on any difference patient/service recipient, general public, community, or stakeholder involvement made to the approach or findings of the study	Not applicable
**Discussion**			
**Study findings, limitations, generalisability, and current knowledge**	26	Report key findings, limitations, ethical or equity considerations not captured, and how these could affect patients, policy, or practice.	Discussion
**Other relevant information**			

Topic	No.	Item	Location where item is reported
**Source of funding**	27	Describe how the study was funded and any role of the funder in the identification, design, conduct, and reporting of the analysis	Acknowledgements, Funding
**Conflicts of interest**	28	Report authors conflicts of interest according to journal or International Committee of Medical Journal Editors requirements.	Acknowledgements, COI

Table S3. Diagnostic accuracy results for a cohort of 10,000 children under 10 years of age with presumptive TB

	Overall			High-risk			Not high-risk		
Scenario 1: TDA-B									
	TB+	TB-		TB+	TB-		TB+	TB-	
Algorithm +	248	4258	4506	144	3665	3809	104	593	697
Algorithm -	59	5435	5494	22	1663	1685	37	3772	3809
	307	9693	10000	166	5328	5494	141	4365	4506
Scenario 2: TDA-A									
Algorithm +	254	3792	4046	149	3261	3410	105	531	636
Algorithm -	53	5901	5954	17	2067	2084	36	3834	3870
	307	9693	10000	166	5328	5494	141	4365	4506
Scenario 3: Stool + TDA-B									
Algorithm +	259	4339	4598	167	3752	3919	92	587	679
Algorithm -	47	5355	5402	10	1624	1634	37	3731	3768
	306	9694	10000	177	5376	5553	129	4318	4447
Scenario 4: Stool + TDA-A									
Algorithm +	264	3890	4154	172	3361	3533	92	529	621
Algorithm -	42	5804	5846	5	2015	2020	37	3789	3826
	306	9694	10000	177	5376	5553	129	4318	4447
Scenario 5: Stool + TDA-B + Referral									
Algorithm +	271	4279	4550	169	3693	3862	102	586	688
Algorithm -	35	5415	5450	8	1683	1691	27	3732	3759
	306	9694	10000	177	5376	5553	129	4318	4447
Scenario 6: Referral									
Algorithm +	913	4418	5331	725	4081	4806	188	337	525
Algorithm -	81	4588	4669	19	2194	2213	62	2394	2456
	994	9006	10000	744	6275	7019	250	2731	2981

Legend for abbreviations: Tuberculosis (TB), treatment-decision algorithm (TDA)

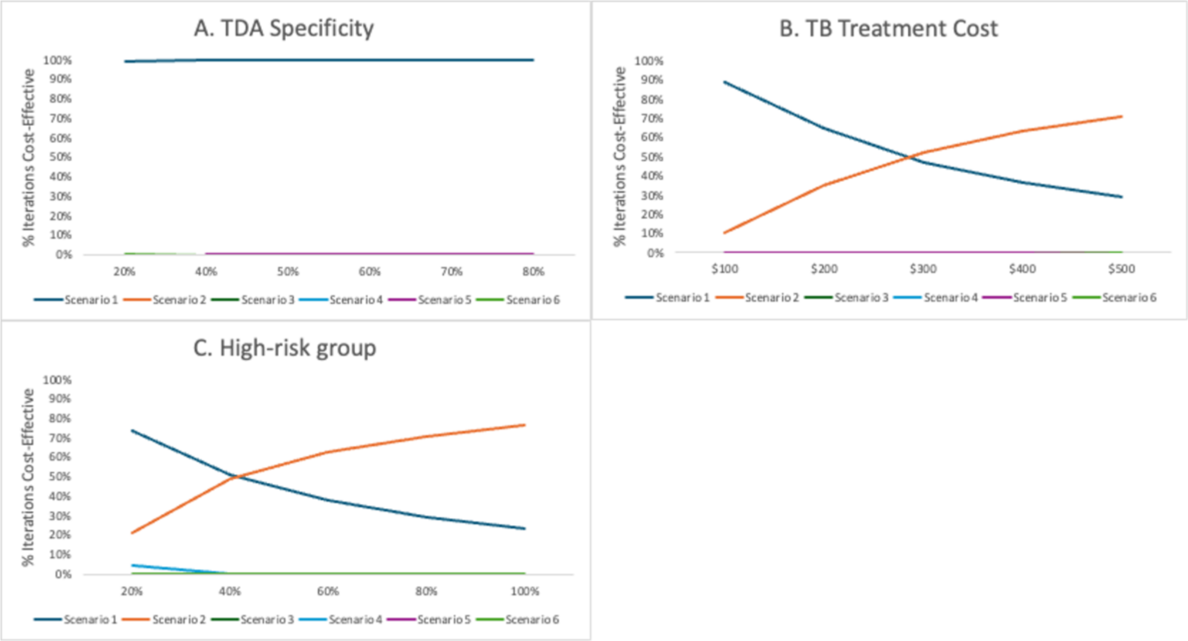

Figure S1: Probabilistic Sensitivity Analyses fixing single parameter values

Legend: Probabilistic sensitivity analyses for the joint uncertainty of parameters using a Monte-Carlo simulation with 10,000 iterations. Parameters with the greatest impact identified from the one-way sensitivity analysis were fixed, as specified in each figure, and the uncertainty of all other parameters were varied. The plots show the percentage of iterations for which scenarios were most cost-effective at the given value of the fixed parameter. In plot A, Scenario 1 (dark blue) remains the most cost-effective as the TDA specificity varies from 20-80%. In plot B, Scenario 1 (dark blue) is most cost-effective at lower TB treatment costs, but Scenario 2 (orange) is more cost-effective as TB treatment costs are greater than $300. In plot C, Scenario 2 (orange) is more cost-effective than Scenario 1 (dark blue) when more than 40% of children are in a high-risk group. In all plots, the other scenarios are cost-effective in at or close to zero percent of the iterations.

References

1. Wobudeya E, et al. Effect of decentralising childhood tuberculosis diagnosis to primary health centre versus district hospital levels on disease detection in children from six high tuberculosis incidence countries: an operational research, pre-post intervention study. EClinicalMedicine 2024:102527 [Epub].
2. Musiime V, et al. HIV prevalence among children admitted with severe acute malnutrition and associated factors with mother-to-child HIV transmission at Mulago Hospital, Uganda: A mixed methods study. PLoS One 2024; 19(4) :e0301887.
3. Bonnet M, et al. Outcome of Children With Presumptive Tuberculosis in Mbarara, Rural Uganda. Pediatr Infect Dis J 2018; 37(2):147-52.
4. Marais BJ, et al. A refined symptom-based approach to diagnose pulmonary tuberculosis in children. Pediatrics 2006; 118(5): e1350-9.
5. Olbrich L, et al. Parallel use of low-complexity automated nucleic acid amplification tests on respiratory and stool samples with or without lateral flow lipoarabinomannan assays to detect pulmonary tuberculosis disease in children. Cochrane Database Syst Rev. 2025; 6: CD016071
6. Seid G, et al. Value of urine-based lipoarabinomannan (LAM) antigen tests for diagnosing tuberculosis in children: systematic review and meta-analysis. IJID Reg 2022; 4:97-104.
7. Gunasekera KS, et al. Development of treatment-decision algorithms for children evaluated for pulmonary tuberculosis: an individual participant data meta-analysis. Lancet Child Adolesc Health 2023; 7(5): 336-46.
8. Nicol MP, et al. Accuracy of a Novel Urine Test, Fujifilm SILVAMP Tuberculosis Lipoarabinomannan, for the Diagnosis of Pulmonary Tuberculosis in Children. Clin Infect Dis 2021; 72(9) :e280-e8.
9. World Health Organization. WHO-CHOICE estimates of cost for inpatient and outpatient health service delivery. Geneva: WHO 2021 Available online from: https://www.who.int/teams/health-systems-governance-and-financing/economic-analysis/costing-and-technical-efficiency/quantities-and-unit-prices-(cost-inputs).
10. Mulogo E, et al. Cost effectiveness of facility and home based HIV voluntary counseling and testing strategies in rural Uganda. Afr Health Sci 2013; 13(2): 423-29.
11. Sun D, et al. Cost utility of lateral-flow urine lipoarabinomannan for tuberculosis diagnosis in HIV-infected African adults. Int J Tuberc Lung Dis 2013; 17(4):552-8.
12. Sekandi JN, et al. Cost-effectiveness analysis of community active case finding and household contact investigation for tuberculosis case detection in urban Africa. PLoS One 2015; 10(2): e0117009.
13. Jo Y, et al. Costs and cost-effectiveness of a comprehensive tuberculosis case finding strategy in Zambia. PLoS One 2021; 16(9): e0256531.
14. Stop TB Partnership. POSEE Sample collection budgeting tool for children. Available online from: http://www.stoptb.org/wg/dots_expansion/childhoodtb/posee.asp.
15. Tucker A, et al. Costs along the TB diagnostic pathway in Uganda. Int J Tuberc Lung Dis 2021; 25(1): 61-3.
16. Gaeddert MJ, et al. A cost-effectiveness analysis of novel stool processing methods for diagnosis of tuberculosis in children under 5 years of age in Uganda. BMC Health Serv Res 2025; (In Press: https://doi.org/10.1101/2025.06.20.25329945).
17. Siapka MV, et al. Cost of tuberculosis treatment in low- and middle-income countries: systematic review and meta-regression. Int J Tuberc Lung Dis 2020; 24(8): 802-10.
